# Molecular profiling of an oleaginous trebouxiophycean alga *Parachlorella kessleri* subjected to nutrient deprivation for enhanced biofuel production

**DOI:** 10.1186/s13068-019-1521-9

**Published:** 2019-07-15

**Authors:** Kashif Mohd Shaikh, Asha Arumugam Nesamma, Malik Zainul Abdin, Pannaga Pavan Jutur

**Affiliations:** 1 Omics of Algae Group, Integrative Biology, International Centre for Genetic Engineering and Biotechnology, Aruna Asaf Ali Marg, New Delhi, 110067 India; 2 Department of Biotechnology, School of Chemical and Life Sciences, Jamia Hamdard University, New Delhi, 110062 India

**Keywords:** Microalgae, Biofuels, Metabolomics, *Parachlorella kessleri*, Nutrient deprivation

## Abstract

**Background:**

Decreasing fossil fuels and its impact on global warming have led to an increasing demand for its replacement by sustainable renewable biofuels. Microalgae may offer a potential feedstock for renewable biofuels capable of converting atmospheric CO_2_ to substantial biomass and valuable biofuels, which is of great importance for the food and energy industries. *Parachlorella kessleri*, a marine unicellular green alga belonging to class Trebouxiophyceae, accumulates large amount of lipids under nutrient-deprived conditions. The present study aims to understand the metabolic imprints in order to elucidate the physiological mechanisms of lipid accumulations in this microalga under nutrient deprivation.

**Results:**

Molecular profiles were obtained using gas chromatography–mass spectrometry (GC–MS) of *P. kessleri* subjected to nutrient deprivation. Relative quantities of more than 60 metabolites were systematically compared in all the three starvation conditions. Our results demonstrate that in lipid metabolism, the quantities of neutral lipids increased significantly followed by the decrease in other metabolites involved in photosynthesis, and nitrogen assimilation. Nitrogen starvation seems to trigger the triacylglycerol (TAG) accumulation rapidly, while the microalga seems to tolerate phosphorous limitation, hence increasing both biomass and lipid content. The metabolomic and lipidomic profiles have identified a few common metabolites such as citric acid and 2-ketoglutaric acid which play significant role in diverting flux towards acetyl-CoA leading to accumulation of neutral lipids, whereas other molecules such as trehalose involve in cell growth regulation, when subjected to nutrient deprivation.

**Conclusions:**

Understanding the entire system through qualitative (untargeted) metabolome approach in *P. kessleri* has led to identification of relevant metabolites involved in the biosynthesis and degradation of precursor molecules that may have potential for biofuel production, aiming towards the vision of tomorrow’s bioenergy needs.

## Background

The global energy demand is increasing day by day as the energy consumption is rising and is expected to increase by 53% within the next two decades. The fossil-derived diesel has been an important source of transportation fuel, but a significant need has come up to look for alternate sources of energy as the conventional source is non-regenerable and costs a lot to the environmental sustainability. The fossil fuel reserves are limited, and as their sources perish, the world will face a huge hike in fuel prices. Since the food and fuel prices are interdependent, the increase in fuel prices will ultimately influence the cost of food [1]. The rapid increase in energy consumption globally has raised the requirement for the development of sustainable renewable energy sources. In the need of current scenario, the production of biodiesel has increased considerably in the recent past with annual production reaching over billions of litres. Mostly waste cooking oil, soybean oil, palm oil, etc. have been used for the production of biodiesel. However, this conventional mode of production, perhaps in the near future, will lead to competition for land usage in terms of fuel and food. Hence, microalgae are being looked upon as a potential source for biodiesel production and have gained considerable attention because of their capability to utilize sunlight and water to convert atmospheric CO_2_ into biomass and biofuels which can prove to be important for both food and energy requirements [2, 3].

Microalgae can produce biomass along with the accumulation of large quantities of lipids/triacylglycerols (TAGs) for biodiesel production. The major advantage for the production of biodiesel from microalgae is their ability to produce large amount of biomass and lipid photosynthetically, and their ability to grow on non-arable land using saline and/or waste waters that make them free from any competition with resources required for growing food [4–7]. Other advantages include their capability to sequester greenhouse gas, a major environmental benefit as the world is facing huge climatic change manifested with conventional fuel utilization [8, 9]; their ability to absorb nutrients from waste waters helping in bioremediation, which is both economical and environment friendly [10, 11]; and their ability to synthesize certain high-value co-products such as OMEGAs, astaxanthin, lutein, tocopherols that are essential for industrial production in pharmaceuticals, nutraceuticals, etc. [12, 13]. One important lead with microalgal-TAG-based biodiesel-derived fuels is their easy integration into the current infrastructure of transportation fuels [14]. Some microalgae can produce TAGs when grown under heterotrophic mode [15], and under autotrophic mode of growth, numerous factors tend to stimulate lipid production such as nutrient availability, light, temperature [16, 17]. Even through microalgae hold potential feedstock for the production of lipids, the accumulation of oil tends to amplify under stress conditions but perhaps the major concern is inhibition of growth, thus simultaneously hampering biomass [18].

Despite such a huge potential microalgae hold for a sustainable source of renewable energy, a number of challenges exist in way for their commercialization as biofuel source. Few microalgal species have been identified as a promising source for industrial-level biofuel, nutraceuticals and pharmaceutical productions, but various research efforts are still being carried out to make microalgal biofuels cost-effective and sustainable. The diverse genera of algae lead to their exceptionally wide range of lipid and metabolic profile which is a result of their dynamic environmental condition [19]. Hence, detailed study on selection, culturing condition optimization, large-scale bioreactor development, bioengineering for better biomass and biofuel, improvement in biomass harvesting and other downstream processing is being carried out to reduce the production cost [20–23]. Several attempts have been made to improve strain performance, harvesting, extraction and culture systems to bring down the economic input for large-scale production [24–28]. The lipid composition among microalgae varies between 10 and 60% (dw) because of the wide range of strains as well as the environmental conditions in which they occur and/or are cultivated [29, 30]. The primary requirement for industrial production of microalgae-based biodiesel is the screening for conditions that induce high lipid productivity in fast-growing microalgae that can fulfil the criteria for sustainable biofuels. Henceforth, in-depth understanding of such phenomenon might also provide deeper insights into the bioengineering of industrially feasible strains. A number of biochemical strategies have been used in this direction to enhance lipid and biomass production [31, 32]. Various environmental factors affect the microalgal cultivation, altering its biomass and biochemical composition [33, 34]. Menon et al. [16] showed that generation of specific intracellular reactive oxygen species (siROS) during stress acts as a common signal that affects various metabolic pathways including lipid biosynthesis. The availability of nutrients affects the microalgal growth as well as their lipid and metabolic compositions [35, 36]. Hence, limiting nutrient availability in the media to induce metabolic variations and lipid accumulation in microalgae is an important alternative strategy to understand the initiation and storage of TAGs in the system.

Despite the significance of various metabolic products in regulating the cellular dynamics, and mechanisms that control the partitioning of these metabolites into distinct carbon-storing molecules in algae, their role in algal physiology and biofuel precursors production is poorly illustrated. In the present work, we have focused on understanding the phenomenon of nutrient deprivation as a tool to enhance lipid productivity as well as the associated changes in the metabolic profiles and biochemical composition of indigenous marine microalga *Parachlorella kessleri* (I) under three different nutrient limitations, viz. nitrogen, phosphorous and sulphur. Previous studies on *P. kessleri* revealed its potential as a suitable candidate for biofuel production, with lipid content around 40–60% of dry cell weight [37–40]. The significance of selecting this indigenous marine microalgae *P. kessleri* (I) is mainly due to its better biomass productivity and higher lipid content as reported earlier [37, 40]. Our rationale highlights up on building a crosstalk between the metabolomic changes and cellular dynamics in terms of biomass and lipid productivities, when this marine microalgae is subjected to nutrient deprivation.

## Results

### Growth and biochemical analysis

**Fig. 1:**
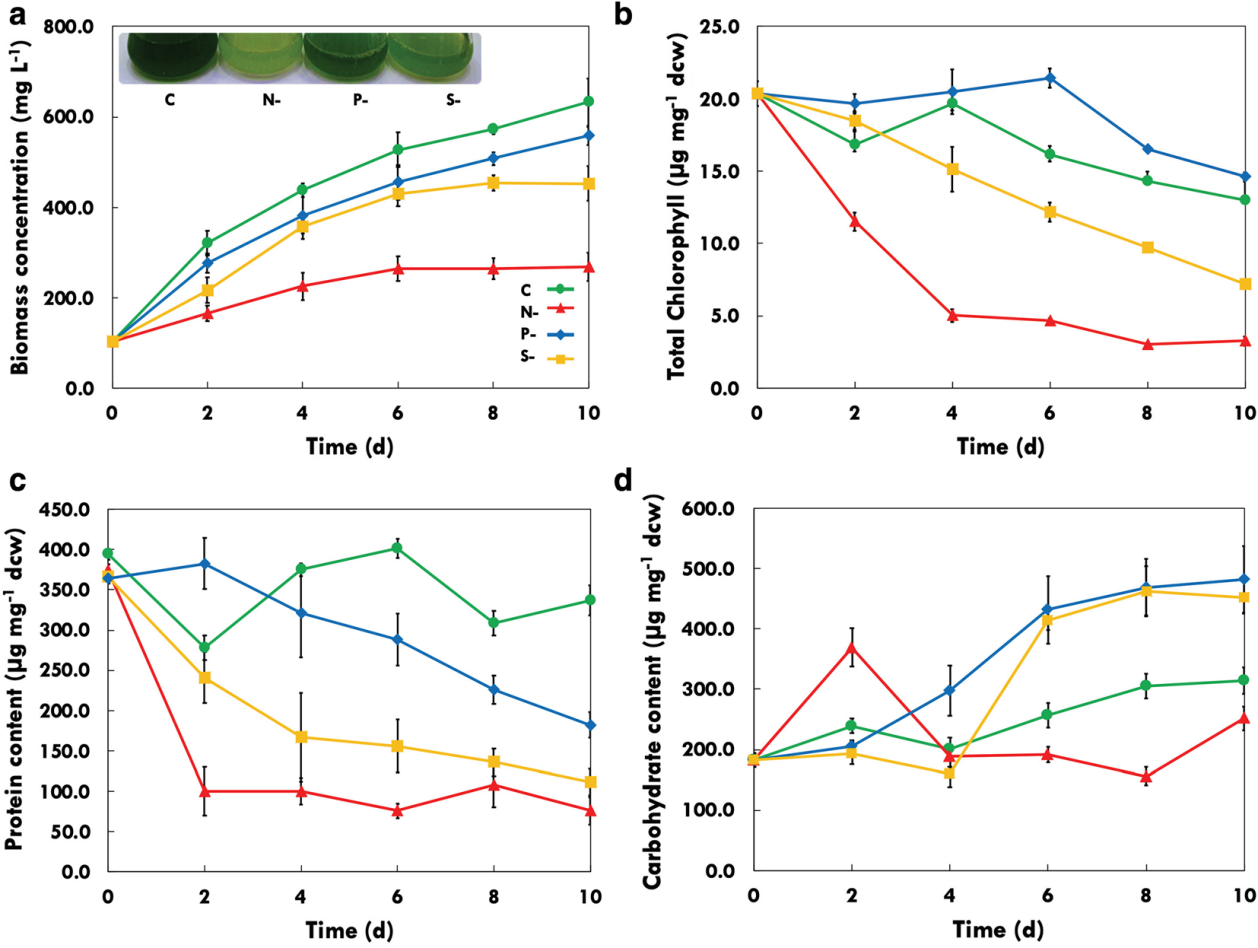
Biochemical profiles of *Parachlorella kessleri* under control and stress conditions. **a** Growth profiles, **b** total chlorophyll, **c** protein and **d** carbohydrate contents; C, control; N-, nitrogen deprivation; P-, phosphorous deprivation; S-, sulphur deprivation; days of treatment—0, 2, 4, 6, 8 and 10 days

The primary impact of nutrient stress is visible on the growth pattern, so the biomass accumulation was analysed for *P. kessleri* under the nitrogen-, phosphorous- and sulphur-deprived conditions. The growth parameters of the marine strain *P. kessleri* under standard growth conditions with an initial inoculum of ~ 0.057 g L^−1^ produced a biomass of 0.54 g L^−1^ in 10 days, thus exhibiting better growth rate, achieving specific growth rate of 0.67 µ and doubling time around 24.7 h. Several studies have shown that microalgae growth depends on an adequate supply of essential macronutrient elements (carbon, nitrogen, phosphorus, silicon), major ions (Mg^2+^, Ca^2+^, Cl^−^, So_4_^2−^) as well as on a number of micronutrient metals such as iron, manganese, zinc, cobalt, copper and molybdenum [41]. To analyse the effect of different nutrient depletions such as nitrogen (N-), phosphorus (P-) or sulphur (S-) on the growth profile, *P. kessleri* was grown under continuous photoautotrophic conditions. The results demonstrated that this strain had severe effect on growth in nitrogen (N-) deprivation, i.e. growth was shunted within 4 days of deprivation after which no change in biomass was observed (Fig. 1a). In sulphur (S-) deprivation and phosphorous (P-) deprivation, no significant change in growth rate was observed till the sixth day (Fig. 1a). In *P. kessleri,* the effect of sulphur (S-) depletion on growth was delayed as compared to nitrogen deprivation. Inset (Fig. 1a) demonstrates the cultures in different deprivation conditions which show growth retardation as well as loss in pigmentation (indicated by pale green colour). The nitrogen concentration in the same medium deficit in N-, P- and S- during the microalgae culture was also estimated. In P- and S- conditions, the nitrogen utilization was slower when compared to the control. Most of the nitrogen was utilized by microalgae at the end of the sixth day in the control, whereas in P- and S- conditions it was completely consumed by the end of the tenth day. This pattern is also observed in the growth profile as the cell growth slows down and shifts towards stationary phase.

Various biochemical constituents were analysed to understand the effect of nutrient starvation over molecular profiling in *P. kessleri*. Figure 1b–d shows the pigment (total chlorophyll), protein and carbohydrate profiles subjected to different nutrient deprivation conditions. The total chlorophyll content was severely reduced under N- stress, while S- deprivation leads to a steady loss of pigments over time. Phosphorus (P-) deprived cells maintained their net chlorophyll levels up to 4–6 days followed by decline in prolonged stress (Fig. 1b). During N- stress, the chlorophyll content was severely deteriorated within 2–4 days of starvation, while P- and S- cells showed a slow decrease. Photosynthetic machinery is the primary component to be affected by severe nutrient deprivation, especially in case of nitrogen deprivation as reported in most of the microalgal strains [42].

The total protein content was also decreased rapidly in case of nitrogen-deprived cells and reached the minimum by the end of the second day. In case of S- deprivation, the decrease was steady over time, whereas in P- stress the protein content started declining after the fourth day (Fig. 1c). During P- and S- conditions, the protein content in the cells declined slowly, but the initial impact on protein machinery was not adverse as seen during N- starvation. When algae are subjected to nutrient deprivation, the cells start to reduce the protein build up and catabolize proteins to use the carbon skeleton to synthesize storage molecules. Figure 1d demonstrates the changes occurring during nutrient stress in the carbohydrate content. Under N- deprived cells, an initial rise in the total carbohydrate content was observed on day 2 (~ onefold), after which it has declined rapidly (Fig. 1d). Our presumption predicts that *P. kessleri* isolated from marine waters may certainly not be a predominant carbohydrate producing strain. Under P- and S- deprivation, an increase in the carbohydrate content was observed till the fourth day. Increase in carbohydrate as a storage molecule has been observed in many algal species, mainly fresh water species, when the cells are subjected to nutrient deprivation.

### Lipid analysis and profiling

**Fig. 2:**
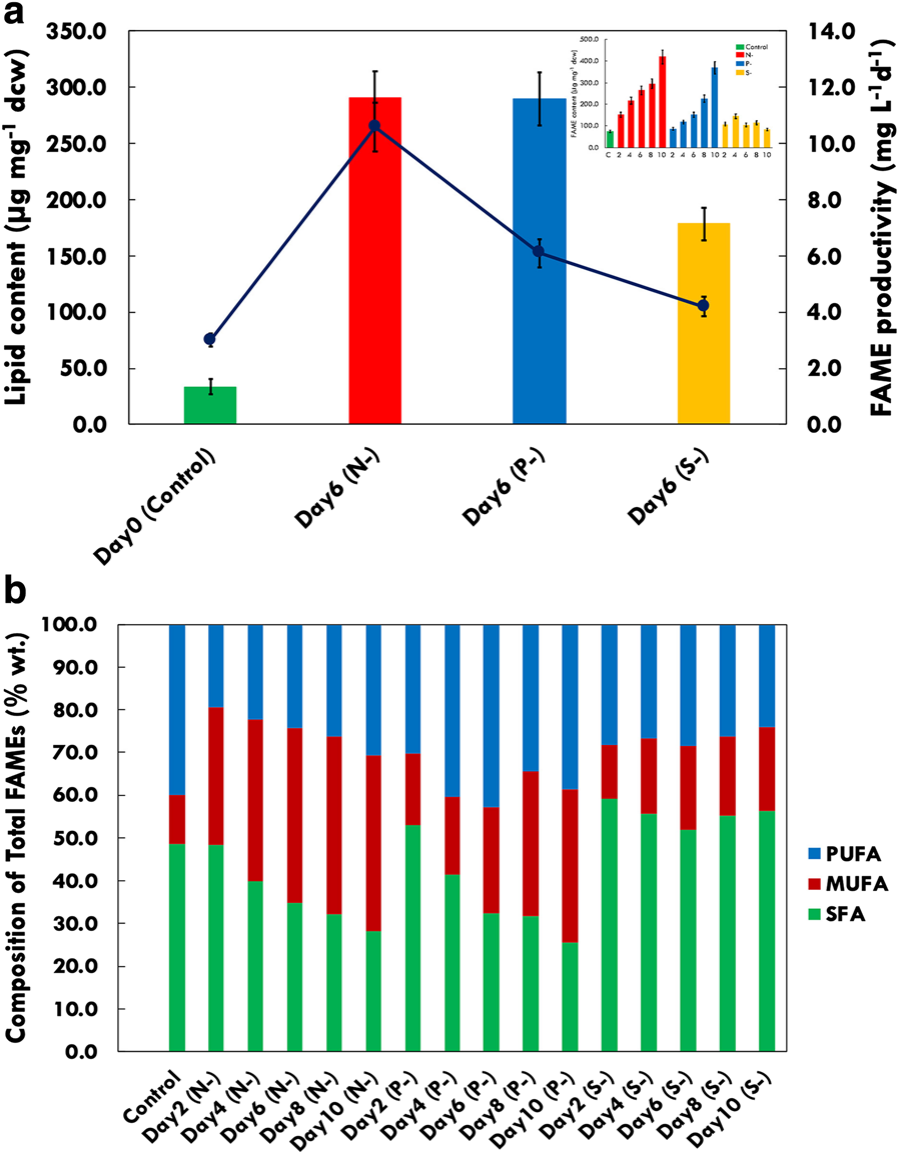
**a** Total lipid content (represented as line graphs) and FAME productivity (in bars) under control and nutrient-deprived conditions for day 6. Inset shows the change in FAME content with stress conditions, days of treatment—0 (control), 2, 4, 6, 8 and 10 days. **b** Changes in saturated (SFA), monounsaturated (MUFA) and polyunsaturated (PUFA) profiles of fatty acids under nutrient-deprived conditions; N-, nitrogen deprivation; P-, phosphorous deprivation; S-, sulphur deprivation; days of treatment—0 (control), 2, 4, 6, 8 and 10 days

Lipid accumulation in algae is induced normally due to environmental stress, particularly when deprived of certain nutrients such as nitrogen, phosphorus, silica, sulphur or certain metals [43, 44]. In microalgae, nutrient deprivation to enhance the production of lipids is a well-observed phenomenon [33, 45]. During stress-induced lipid accumulation, the lack of essential nutrients such as N, P and S restricts the capacity of cellular division as a result of which the organism shifts towards alternative pathways for inorganic carbon assimilation, thus shuffling the carbon towards the storage biomolecules, i.e. TAGs. To demonstrate the effect of different nutrient deprivation on the synthesis of TAGs, total lipid was extracted from cells and analysed using thin-layer chromatography (TLC) as described in “Methods”. The TLC plate loaded with extracted lipids from samples of the three stress conditions (N-, P- and S-) led to a sharp increase in the TAG content in *P. kessleri*, where TAG seems to increase with the progression of duration of starvation (data not shown). Further, the samples were quantified using GC–MS analysis to evaluate the lipid productivity subjected to nutrient stress. Sulphur (S-) depletion induced TAG formation to a much lesser extent as compared to other nutrient-deprived conditions. The increase in TAG content can be observed from the second day itself in N- condition, whereas in P- and S- depletion TAG accumulation was observed after the fourth day (Fig. 2a). In P- depletion, a gradual increase in TAG content was observed without compromising cell growth. Under N- stress, the TAG production has been initiated on day 2 itself and reached maximum by the tenth day, but also lead to severe growth inhibition depicting metabolic changes within the cells. In marine microalgae *P. kessleri*, S- depletion lead to inhibition in growth after day 6 but in comparison with N- and P-, the increase in lipid content was not very significant (Fig. 2a).

A number of changes occur in the overall neutral lipid content as well as the saturation profile of lipids when microalgae are subjected to nutrient deprivation [46]. Figure 2a, b shows the lipid content (µg mg^−1^ dw) and FAME productivity (mg L^−1^ day^−1^) analysed through GC/MS under different nutrient (N, P and S) deprivation conditions compared to the control in *P. kessleri*. Our data show that FAME productivities were similar in N- (~ 11.63 mg L^−1^ day^−1^) and P- (~ 11.58 mg L^−1^ day^−1^), while lower in case of S- deprivation (~ 7.13 mg L^−1^ day^−1^) at the end of the sixth day (Fig. 2a). As compared to nitrogen and phosphorous depletion, the FAME productivity under sulphur limitation was substantially low (Fig. 2a).

The FAME content per cell seems to be higher in N- stress (Fig. 2a), where a constant increase in lipid accumulation was observed, i.e. reaching up to > 40% of dry cell weight, while in P- the lipid content per cell remains low as compared to N- cells but is considerably higher than control (Fig. 2a inset). Overall, the time-dependent changes in lipid content for 10 days in different stress conditions are shown in Fig. 2a (inset). In the present study, *P. kessleri* subjected to N- has shown enhanced neutral lipid content, whereas P- tends to have a steady increase (Fig. 2a).

On initial day (control) of inoculation, FAME profiling patterns showed highest content of polyunsaturated fatty acids (PUFAs) and saturated fatty acids (SFAs) than monounsaturated fatty acids (MUFAs) (Fig. 2b). Under N- conditions, *P. kessleri* exhibits an increase in MUFAs (up to 41% of total FAME) with considerable decrease in SFAs (to ~ 35%) and PUFAs (to ~ 24%) by the end of the tenth day. This can be a result of the oxidative damage to PUFAs under stress [47] or recycling of membrane lipids towards TAGs [48]. Although P- limitation induced lipid biosynthesis, at the end of deprivation period, the FAME pattern suggests decrease in SFAs (to ~ 32.5%) and considerable increase in MUFAs (to ~ 24.5%) and PUFAs (to ~ 43%). In S- depleted cells, SFAs increased (to ~ 52%) with a little upregulation in MUFAs (to ~ 20%) while PUFAs decreased (Fig. 2b).

### Metabolome analysis

**Fig. 3:**
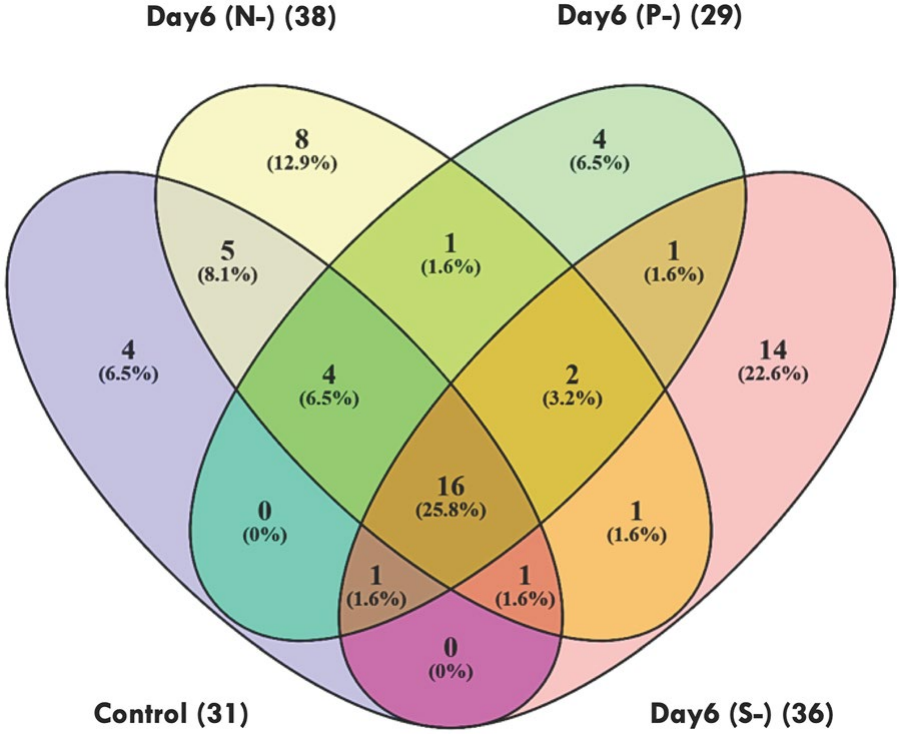
Venn diagram representing various metabolites in different stress conditions. C, control; N-, nitrogen deprivation; P-, phosphorous deprivation, S-, sulphur deprivation. Numbers in brackets show total metabolites obtained in each condition

Metabolite levels are tightly controlled during the starvation condition to enhance the chances of survival. A number of changes were observed in terms of growth and biochemical profiles under different nutrient starvation conditions. Therefore, to understand the molecular profiling, we have employed qualitative metabolomics tool to evaluate the changes occurring during stress which will provide new insights for enhancing the lipid production. The metabolite extraction and derivatization were carried out in all the samples of *P. kessleri* as described in “Methods” section. A total number of ~ 62 metabolite peaks were obtained after manual curation and analysis of raw data. The most common phenomenon observed in the raw data files is the repetition of same metabolite as a result of alternate derivatization [36]; such metabolites were removed if not significant. All the metabolites analysed in *P. kessleri* under different nutrient deprivation were plotted using Venny 2.1 (http://bioinfogp.cnb.csic.es/tools/venny/) to find out intersecting and differential metabolites (Fig. 3). Among these, eight metabolites were exclusively expressed under N-, four in P- and 14 in S- conditions (Fig. 3).

**Fig. 4:**
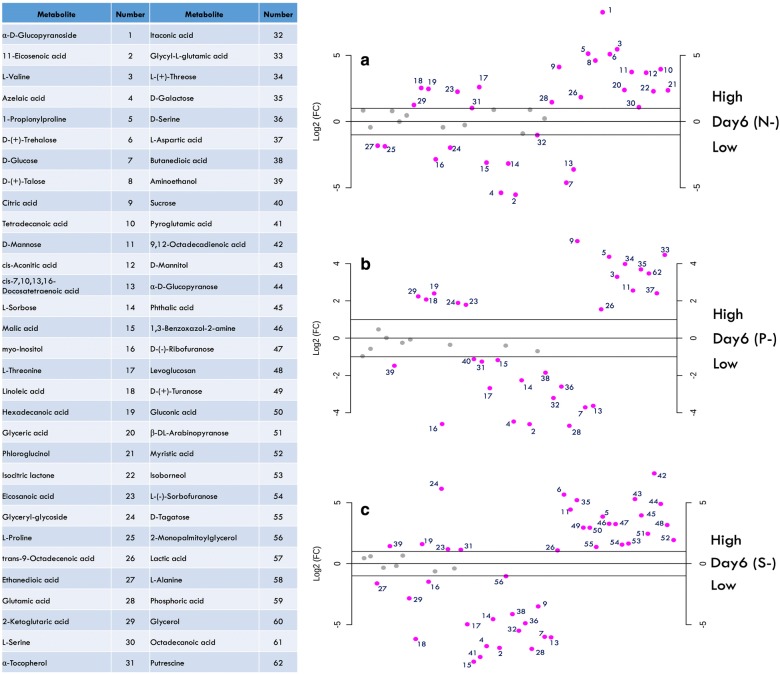
Fold-change in metabolites under stress conditions; **a** nitrogen deprivation: N-, **b** phosphorous deprivation: P-, **c** sulphur deprivation; table represents numerical abbreviations of the metabolites

Certain metabolites were common in all stress conditions, while certain were common in two conditions, as discussed later. The fold change for all the metabolites subjected to stress conditions either upregulated or downregulated as compared to the control is shown in Fig. 4a–c. (The list for metabolites with their representative numeric code is included.) In N- cells, many metabolites such as valine, trehalose, citric acid, mannose, linoleic acid, trans-9-octadecanoic acid, talose were found to increase > twofold, while malic acid, myo-inositol, glucose, polyunsaturated fats were predominantly decreased (Fig. 4a). In P- cells, upregulated metabolites include citric acid, galactose, mannose, threose, while myo-inositol, glucose, azelaic acid, sorbose, a-tocopherol were decreased (Fig. 4b). In S- cells, metabolites such as trehalose, mannitol, galactose, mannose were increased, while malic acid, glutamic acid, citric acid, myo-inositol decreased (Fig. 4c).

**Fig. 5:**
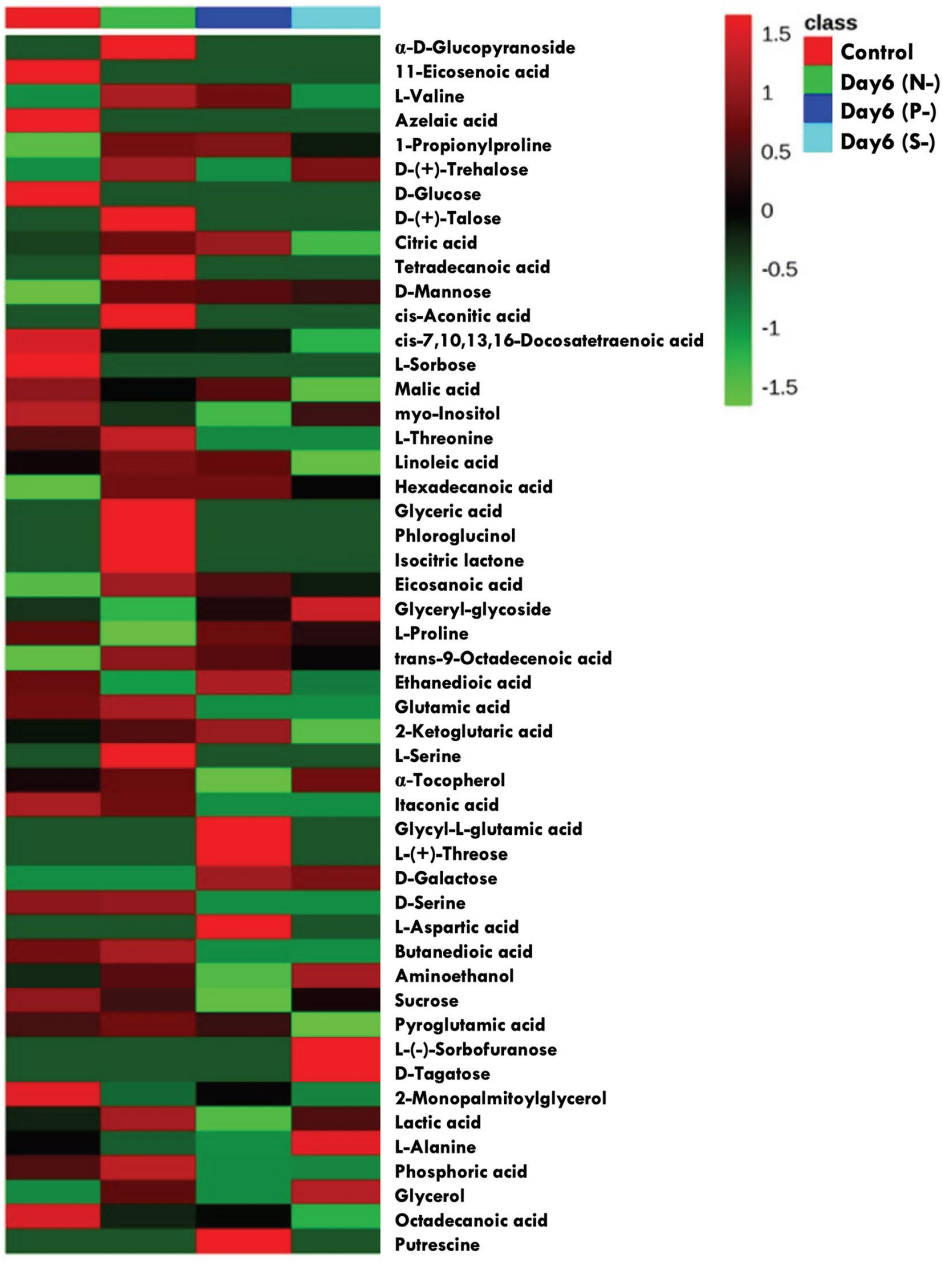
A heat map illustrating the expression of all the metabolites under nutrient deprivation conditions. N-, nitrogen deprivation; P-, phosphorous deprivation; S-, sulphur deprivation

The overall metabolomic profiles in *P. kessleri* when subjected to nutrient deprivation have been illustrated as a heat map for the visualization of expression profiles of various metabolites (Fig. 5).

**Fig. 6:**
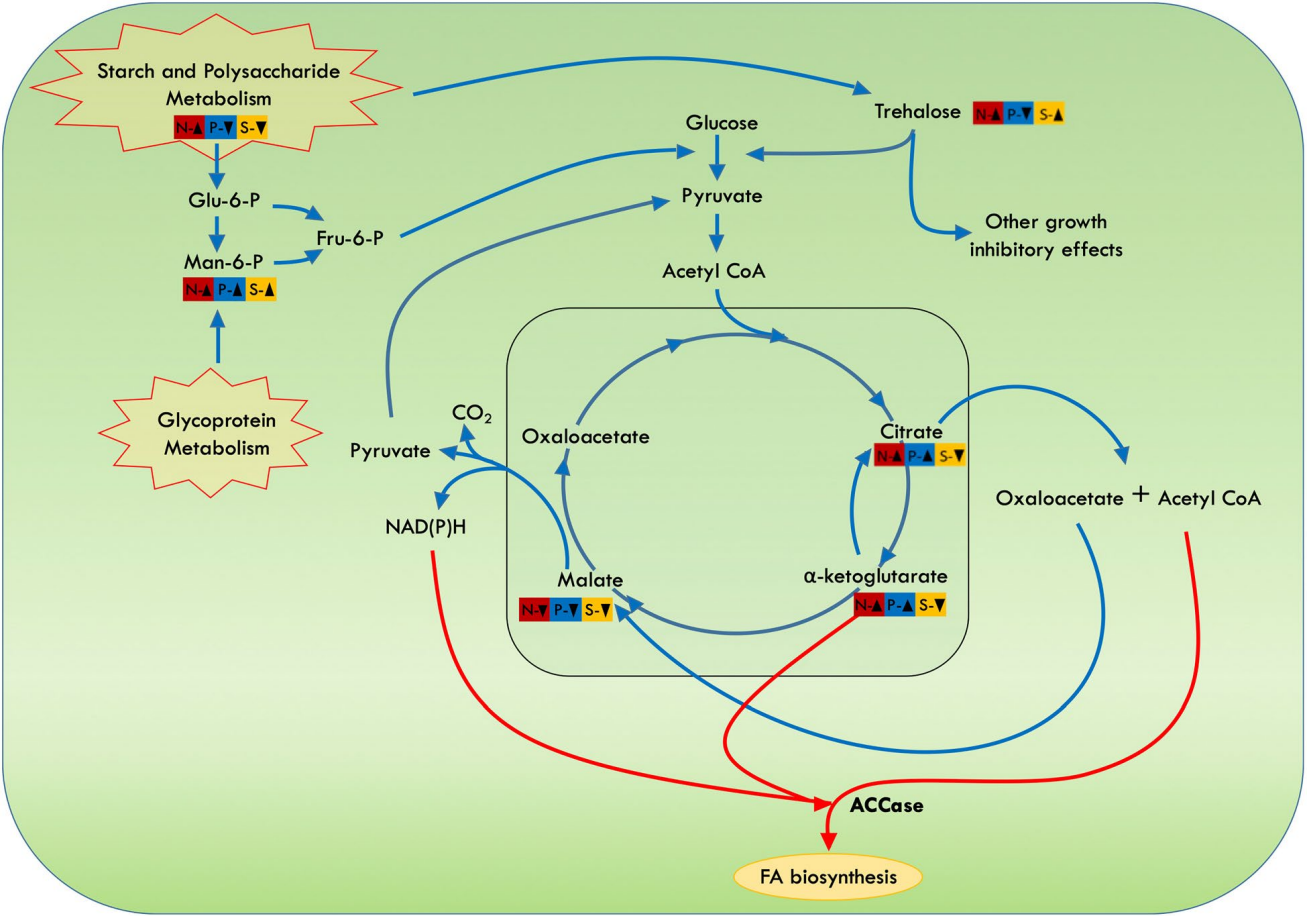
Schematic representation of metabolic pathway reactions altered under stress; colour codes for different stress treatments. Red arrows show pathways routing towards fatty acid (FA) biosynthesis. Upregulation (↑); downregulation (↓)

An essential metabolite of interest, citric acid, was found to be upregulated nearly by fivefold in only N- and P- conditions. It seems to be an important metabolite in upregulating the FA biosynthesis as it increased in both N- and P- conditions where lipid accumulation has also increased, while it decreased in S- where lipid accumulation is much lower as compared to other two conditions. Also, 2-ketoglutaric acid was found to increase in the similar conditions by twofold, both together assume to divert flux towards FA biosynthesis [49]. Another metabolite of importance is trehalose that was found enhanced fivefold in N- and S- but not in P- conditions that may presumably play a significant role in cell growth regulation. Significant metabolite changes occur when subjected to nutrient depletion (N-, P- and S-) conditions, and each of these changes will affect cell growth and lipid productivities. Henceforth, our metabolomic data provide us with the schematic model to understand flux diversion that leads to changes in lipid productivity and growth rate under different nutrient stresses (Fig. 6).

## Discussion

Growing bioenergy needs demand urgent action to generate renewable fuels at feasible cost. Algae seem to be a promising bioresource in terms of economically feasible bioenergy producer, yet the information regarding cellular dynamics of microalgal cells is fairly poor. The primary focus in algal research has been the enhancement of lipid production employing nutrient stress while biomass productivities are essentially compromised. A number of strategies, such as nutrient deprivation, light intensity, temperature variation, carbon dioxide have been employed to reach specific goals, but the cellular behaviour under these conditions is yet not well established. Under the adequate supply of nutrients including C, N, P, S and light, depending on the strain selection, the biomass productivity can be high but the lipid content seems to be as low as 5% w/w or even less [50]. During unfavourable conditions, the microalgal growth and photosynthetic activity cease, while the excess energy gets assimilated as lipids and/or carbohydrates. Metabolically, there is always a competition between biomass and storage molecule assimilation, which governs and channelizes the carbon flux either towards biomass accumulation or towards lipid and carbohydrate synthesis. Depending on the requirement, a metabolic shift can switch the photosynthetic assimilation of inorganic carbon from biomass synthesis to energy storage metabolism [51, 52]. Changes in the environmental conditions such as light intensity, nutrient limitation, salinity, temperature, pH, and culture age invariably affect the lipid content of microalgae [53–59]. Oleaginous microalgae can utilize their lipid metabolic pathway for the biosynthesis and accumulation of lipids in the form of triacylglycerols (TAGs) [60]. Some of these oleaginous microalgae can even store TAGs up to 40% to 70% of their dry weight [33, 61]. These lipids are typically storage reserves within the cell that helps the organism to sustain adverse environmental conditions. Henceforth, the competition in terms of biomass (or) lipid accumulation depends upon the different levels of perturbation [62]. In the present work, growth and cellular physiology of *P. kessleri* were demonstrated under different nutrient deprivations. While growth was severely hampered under N- deprivation, the cells were able to withstand phosphorus or sulphur absence fairly well, although after 6 days the S- cells showed high decline in growth. Perhaps the nutrient deprivation can be attributed to the evolutionary behaviour of microalgae, for example in marine waters the phosphorous availability is quite dynamic, and hence, these photosynthetic organisms do have specialized responses to maintain their growth under certain nutrient depletion conditions [63, 64]. Nitrogen, a major component of proteins, enzymes and nucleotides, is extremely essential, without which growth cannot be sustained.

A number of physiological changes are observed when microalgae are subjected to nutrient deprivation. During stress, the cell machinery will try to minimize the protein synthesis due to non-availability of nitrogen by shutting down the protein biosynthesis and degrading the protein pool to amino acids to get energy for survival as well as to assimilate carbon as storage molecules. Under nutrient-deprived condition, the total chlorophyll content decreased as the days of starvation progressed. Under nitrogen-deprived condition, the chlorophyll content was severely deteriorated within 2 to 4 days of starvation, while phosphorous- and sulphur-starved cells showed a slow decrease. Chlorophyll reduced to almost half within 2 days of nitrogen starvation (Fig. 1b). This is a very common phenomenon observed under nitrogen stress among other strains too. The protein content did show variable response to different stress conditions. Under nitrogen deprivation, rapid lowering in protein content is observed. The cell machinery tries to cope up with nitrogen unavailability by cutting down on protein synthesis and degrading the protein pool to amino acids to get energy for survival as well as to divert carbon towards storage molecules. Phosphorous- and sulphur-starved cells also showed a slow decline in the protein content, but the initial impact on protein machinery was not as severe as nitrogen starvation Fig. 1c). A sharp increase in carbohydrates in nitrogen-starved cells was observed on the second day, where the carbohydrate content was doubled, which later decreased substantially. This initial response might be attributed to the formation of carbohydrates as energy storage molecule in the case of extreme nitrogen limitation, which later provides carbon skeleton for lipid biosynthesis as storage molecules under prolonged starvation. The increase in carbohydrate content in phosphorous- and sulphur-starved cells was observed after day 4, and the lipid accumulation was observed late as compared to nitrogen-starved cells (Figs. 1c, 2a). These observations suggest that the microalgae initially store carbohydrates energy reserves to overcome the initial stress and then utilize the same for energy generation when they are subjected to severe macronutrient limitation. This has been reported in various microalgae, where some accumulate starch under nutrient depletion condition, others accumulate lipids, or an initial starch accumulation followed by lipid accumulation over prolonged stress is observed [65–69]. In conclusion, adverse effects in molecular profiles of biomolecules (such as total chlorophyll, proteins and carbohydrates) were seen during N- and S- conditions with hampered cell growth (biomass), whereas P- stress seems to have a limited effect.

Under optimal conditions, photosynthesis and electron transport chain produce ATP and NADPH which is utilized as energy currency during cell division [70]. Hence, the optimal ratio of reduced and oxidized metabolites is maintained, whereas during nutrient deprivation the pool of NADP+ and ADP depletes as photosynthesis continuously produces NADPH and ATP which remains under-utilized [62]. Biosynthesis of fatty acid consumes NADPH and ATP; hence, the increased fatty acid synthesis helps the cells to maintain the balance of required electron acceptors (NADP+). During nutrient limitation, an increase in the lipid content has been demonstrated in several microalgal species [38, 63, 71]. Nitrogen is an essential requirement for protein synthesis as well as photosynthesis, but under nitrogen-limiting conditions most of the carbon fixed in photosynthesis is channelized towards the production of lipids and/or carbohydrates. Several studies have demonstrated that nitrogen deprivation leads to higher accumulation of lipids in various microalgal strains [72, 73]. Upon removal of nitrogen, *Nannochloropsis* sp. and *Neochloris oleoabundans* increased their lipid content to onefold and twofold, respectively [74, 75]. A number of cellular metabolic processes such as photosynthesis, signal transduction, energy transport system require phosphorous as the main component, and hence, the deficiency of this major element also results in accumulation of lipids [76–78]. Similar studies showed increase in lipid accumulation to more than 50% of dry weight (dw) under P- limitation in *Scenedesmus* sp. LX1 belonging to *Scenedesmaceae* [79, 80]. In our present investigation, FAME content in *P. kessleri* was highest in N- cells, whereas in P- cells the same has been compensated by the better growth rate. This observation was unique as the cells were able to produce lipids without compromising growth and the mechanism is still to be exploited. Further investigation can provide us with novel insights for enhancement of lipids among specific strains without negotiating growth. However, the FAME profiling depicting the saturation and unsaturation levels was more promising in the case of N- cells. Nitrogen-deprived cells had a high level of SFAs and MUFAs, while PUFAs levels are decreased (Fig. 2b). This profile is more suitable in terms of biodiesel as the amount of polyunsaturation affects overall properties of biodiesel. Lower PUFA level is mainly helpful in lowering ignition delay, increasing stability against oxidation and lowering NOx emission [81], whereas in P- cells the PUFA content was higher than SFAs and MUFAs in *P. kessleri*.

A number of studies on *Chlamydomonas* have shown a strict metabolite regulatory network balancing the cellular processes under stress [82–84]. Various chromatographic techniques are used for the identification and analysis of metabolites from biological samples. Gas chromatography combined with mass spectroscopy (GC–MS) has become a popular technique to analyse metabolomic phenotypes, where GC separates the volatile compounds depending on their mass and polarity, while MS enables reproducible analyte fragmentation and identification [85, 86]. The metabolomic analysis of the cells subjected to nutrient starvation has shown a number of metabolites, which had low match scores and/or duplicated because of the varying degree of sialylation. A total number of 62 metabolites were screened and analysed on the basis of their relative peak areas from deprived conditions. Since different conditions gave different metabolic profiles, we tried to explore those that can give a probable crosstalk among the various stresses. Overall, the downregulated metabolites were more or less common among all conditions, such as malic acid, sorbose, glucose, myo-inositol (Figs. 4, 5). Also, sugar molecules obtained from polysaccharide degradation usually increased in starved cells, indicating a cut down of stored carbohydrates to provide carbon skeleton for lipid synthesis. In all stress conditions, two metabolites, i.e. 1-propionyl proline and mannose, were found to be upregulated compared to the control. Martel [87] reported increase in mannose, a C-2 epimer of glucose, which can be derived from the digestion of polysaccharides and glycoproteins under the nitrogen-deprived condition in *Isochrysis galbana*. However, in the present study increase in mannose seems to be higher in the N- condition due to cumulative breakdown of polysaccharides and glycoproteins, whereas in P- and S- stresses it may be due to glycoprotein metabolism alone. The predominant fatty acids depicted in the analysis are hexadecanoic acid, eicosanoic acid (arachidic acid) and trans-9 octadecanoic acid (elaidic acid) (Figs. 4, 5). Myo-inositol, a sugar alcohol, has also been reduced many folds under all the stress conditions. Inositol is an important component of structural lipids and may get disrupted during stress. In plants, the hexophosphate of inositol serves as a phosphate reserve [88] and the same mechanism may also be present in these microalgae as well because of the decreased content of myo-inositol in P- cells as compared to N- or S- cells. Henceforth, phosphate derivatives of myo-inositol might be broken down to provide phosphorous for cellular activities under P- deprivation. Also, sorbose, another monosaccharide, and azelaic acid also seem to be utilized for cell survival in *P. kessleri*. Malic acid, an intermediate of tricarboxylic acid (TCA) cycle, is decreased during stress and predicts the downplay of the Calvin cycle. The decarboxylation of malate to pyruvate leads to the generation of NAD(P)H, and both the pyruvate and NAD(P)H can be utilized for FA biosynthesis leading to lipid accumulation [89]. During C_4_ carbon fixation in plants, malate provides CO_2_ to Calvin cycle and such similar mechanism may co-exist in some marine diatoms and algae [90, 91]. In *P. kessleri*, the reduced photosynthetic machinery might also lead to the break down of malate as it will not be required to transport CO_2_. α-Tocopherol was also enhanced in nitrogen and sulphur deprivation. α-Tocopherol is another potent antioxidant that is enhanced under stress to protect cells from oxidative damage by quenching reactive oxygen [92] and also involved in the regulation of photosynthesis and macronutrient uptake and utilization [93].

Citric acid positively regulates acetyl-CoA-carboxylase which is the enzyme for the first committed step towards FA biosynthesis [94]. Citric acid is an intermediate of Calvin cycle; when transported from mitochondria to cytoplasm, it breaks down into oxaloacetic acid and acetyl-CoA diverting flux towards FA biosynthesis. Increased citrate may act as an acetyl-CoA carrier for fatty acid synthesis [95]. In *Nannochloropsis salina*, kinetic profiles and activity studies showed that this Eustigmatophyceae strain is able to convert sugar via citrate cycle towards lipids [96] and the exogenous supply of citrate showed increased fatty acid biosynthesis in *Chlamydomonas* sp. [85]. Upregulated citrate may provide acetyl-CoA in cytoplasm which can further be utilized to produce FA molecules. Citric acid was highly upregulated in both N- and P- cells. Similarly, 2-ketoglutaric acid has also emerged as a master regulator in essential pathways. Like citric acid, it is also a cataplerotic molecule, enhancing to provide synthesis and regulation of other molecules desired by the cells. It was found to be interacting with the regulator of acetyl-CoA carboxylase enzyme (ACCase), thus relieving ACCase for fatty acid biosynthesis [49]. 2-Ketoglutaric acid was also found to increase in nitrogen stress, providing backbone for nitrogen assimilation [97]. Amino acid degradation may also lead to accumulation of 2-ketoglutarate, which can be further converted back to citrate for FA synthesis [98].

Trehalose was found to be upregulated in nitrogen- and sulphur-deprived cells. Trehalose is a non-reducing disaccharide that performs a variety of functions, from carbon storage to carbon metabolism, protection from osmotic stress, stabilization of membranes and proteins, removal of aberrant storage material, protection from oxygen radicals, induction of autophagy [36, 85, 99]. The induction of trehalose might be responsible for growth retardation in N- and S- conditions. Previous reports also observed an increase in trehalose under nitrogen starvation in *Chlamydomonas* after 6 days of depletion [85]. Trehalose may also control various metabolic processes and growth [100]. It may act as a growth regulator by affecting hexokinase and thus glycolysis, and leads to severe growth defects such as dwarfism in plants [101, 102]. Although sulphur deprivation does not show growth inhibition initially, later growth shunts probably because of increase in trehalose accumulation. This seems interesting as the presence of citric acid and 2-ketoglutaric acid in nitrogen and phosphorous starvation might induce lipid accumulation, while trehalose presence in nitrogen and sulphur depletion might result in growth retardation (Fig. 6). As a result, a crosstalk between these metabolites such as citric acid, 2-ketoglutaric acid and trehalose might be important for the production of biomass as well as lipid accumulation in marine microalgae *P. kessleri*.

## Conclusions

*Parachlorella kessleri* subjected to nutrient deprivation shows growth retardation except under P- limitation. Nitrogen and phosphorous limitation played a major role in lipid accumulation. The qualitative metabolomics showed a variable shift in the metabolite flux in response to different stress conditions. A crosstalk between metabolites, namely citric acid, 2-ketoglutaric acid and trehalose, can be hypothesized to have greater impact on the production of biomass and lipid accumulation. To our knowledge, this report in the marine microalgae *P. kessleri* is a new paradigm to elucidate the molecular changes in the basis of metabolite redistribution subjected to nutrient-limiting conditions leading to insights on the production of biomass, biofuels and bioproducts (B^3^) in non-model systems. In conclusion, marine strain *Parachlorella kessleri* with high biomass and higher lipid productivity was analysed where shuffling of certain metabolites when subjected to stress will dictate the profile changes that may prove to be a benchmark for over-expression of lipids without compromising growth. Further characterization of this strain may be a critical step towards making algae-derived biofuels economically competitive for industrial production.

## Methods

### Microalgae and culture conditions

Marine microalgae *P. kessleri* (I) (procured from Indian Institute of Technology-Madras, Chennai) was grown in minimal media F/2 [103] under constant illumination (~ 100 µmol m^−2^ s^−1^ photosynthetically active radiation [PAR]) on an orbital shaker at 150 RPM at 25 °C. The composition of media components for F/2 media (g L^−1^) is as follows—NaNO_3_—0.075; NaH_2_PO_4_·2H_2_O—0.005; Na_2_SiO_3_·9H_2_O—0.03 in artificial sea water (ASW) prepared using NaCl—24; MgCl_2_·6H_2_O—11; Na_2_SO_4_—4; CaCl_2_·6H_2_O—2; KBr—0.1; H_3_BO_3_—0.03; Na_2_SiO_3_·9H_2_O—0.005; SrCl·6H_2_0—0.04; NaF—0.003; NH_4_NO_3_—0.002; Fe_3_PO_4_·4H_2_O—0.001; trace metals solution (in g L^−1^)—1 mL L^−1^ [ZnSO_4_·7H_2_O—0.023; MnSO_4_.H_2_O—0.152; Na_2_MoO_4_·2H_2_O—0.007; CoSO_4_·7H_2_O—0.014; CuCl_2_·2H_2_O—0.007; Fe(NH_4_)2(SO_4_)_2_·6H_2_O—4.6; Na_2_EDTA·2H_2_O—4.4]; and vitamin B_12_*—0.135 mg L^−1^; biotin vitamin solution*—0.025 mg L^−1^; thiamine vitamin solution*—0.335 mg L^−1^ (*added after autoclaving the media). Growth and biomass accumulation were monitored by cell count using haemocytometer [104] and dry weight (dw) analysis as described previously [105]. Growth rates were obtained using the following equation [106]

1 $$K = \frac{{\ln \frac{{N_{2} }}{{N_{1} }}}}{{t_{2} - t_{1} }}$$

where *N*_1_ and *N*_2_ represent cell counts at initial time (*t*_1_) and final time (*t*_2_), respectively. Doubling time was calculated depending on the specific growth rate [107].

2 $${\text{Doubling}}\;{\text{time}} = \frac{\ln 2}{K}.$$

Cells were initially grown photoautotrophically to the middle of the logarithmic phase in F/2 medium. These cells were collected by centrifugation and resuspended again at a density of 2 × 10^6^ cells mL^−1^ in regular F/2 or in the same media completely deficit in nitrogen (N-), phosphorous (P-) or sulphur (S-). Nitrogen concentration in the media was estimated during culture growth spectrophotometrically as described by Yodsuwan et al. [108]. Samples for all the analyses were taken immediately after resuspension (control, 0 day) and at the time intervals of 2, 4, 6, 8 and 10 days for further experiments, and the sixth-day samples were analysed for metabolomic profiling.

### Biochemical analysis

The samples were analysed for changes in the biochemical constituents [pigments (total chlorophyll), proteins, carbohydrates] subjected to the nutrient stress. For estimation of pigments, 1 mL of culture was pellet down and resuspended in 1 mL of absolute methanol. The suspension was vortexed briefly and incubated at 4 °C for an hour to extract the pigments completely. The debris was pellet down, and the suspension was used to measure absorbance at 665, 652 and 470 nm to calculate total chlorophyll content [109]. Protein estimation was done using modified biuret method. Total soluble proteins were extracted using 1 N NaOH in 25% methanol as extraction buffer. 1–2 mL of culture was pellet down and resuspended in 1 mL of extraction buffer and incubated at 80 °C for 15 min. The sample was cooled down to room temperature and centrifuged at high speed to remove debris. One hundred microlitres of extract was mixed with 50 µL of CuSO_4_ solution (0.21% CuSO_4_ in 30% NaOH), incubated at RT for 10 min and its absorbance was measured at 310 nm [110]. Carbohydrate estimation was done using modified phenol–sulphuric acid method. Around 100 µL of cells was taken, and absolute H_2_SO_4_ was added and kept for 1 h at room temperature. Afterwards, 5% phenol was added along with 1 mL of H_2_SO_4_ and kept at room temperature for another 20 min after vortexing. Absorbance was measured at 490 nm [111].

### Lipid quantification and profiling

Total lipids were extracted using modified Bligh and Dyer procedure [112], dried under N_2_, and visualized as TAGs by thin-layer chromatography (TLC) on a silica gel plate. Briefly, ~ 1 × 10^8^ cells were collected in a glass tube with Teflon-lined screw cap. Lipid extraction was done using methanol/chloroform (2:1, v/v) containing 0.01% butylated hydroxytoluene. Two millilitres of methanol/chloroform mix was added to the cell pellet and incubated at 25 °C for 2 h with shaking. Thereafter, chloroform (1 mL) and water (1.8 mL) were added to the tubes, mixed vigorously, and centrifuged at 3000×*g* to separate the mix into two phases. The lower organic phase containing the extracted lipids was transferred to a new glass tube with the help of a Pasteur pipette. Extracted organic phase was dried at 50 °C under stream of nitrogen (N_2_) to evaporate the solvent completely and resuspended in CHCl_3_/MeOH (100 µL, 6:1 v/v). Fifty microlitres of this extract was applied to a silica 60 thin-layer chromatography plate (Sigma-Aldrich) and run with a solvent system of heptane/ethanol/acetone (70:30:1, v/v/v) to resolve the neutral lipids. The TAG band was identified by staining co-migrated TAG standard with iodine vapours [84]. For GC–MS analysis, ~ 1 × 10^8^ cells were acid-hydrolysed and methyl-esterified using 2% sulphuric acid in methanol (300 µL) for 2 h at 80 °C. Prior to the reaction, 50 µg of heptadecanoic acid (Alfa Aesar) was added as internal standard. The fatty acid methyl esters were extracted using 300 µL of 0.9% (w/v) NaCl solution and 300 µL of hexane. The mixture was vortexed briefly and centrifuged at 3000×*g* for 3 min to separate the phases. One microlitre of hexane layer was injected into a 7890A gas chromatography (GC) mass spectrometry (MS) system equipped with a 7000 GC/MS triple quadrupole system (Agilent) [107, 113]. The running conditions for GC–MS were described by Agilent’s RTL DBWax method [114].

### Qualitative metabolomics

For the extraction of cellular metabolites, ~ 10^9^ cells were collected by centrifugation at 8000×*g* for 10 min and immediately quenched in liquid nitrogen. Metabolites were extracted using methanol, chloroform and water by repeated freezing and thawing. Cells were resuspended in 1 mL of ice-cold methanol/chloroform/water (10:3:1) and vortexed briefly. The cells were frozen again in liquid nitrogen for 1–2 min and thawed on ice for 4–5 min. Freezing and thawing cycles were repeated five times with intermittent vortexing. Samples were then centrifuged at 14,000×*g* for 3 min at 4 °C to get rid of cell debris. The supernatant was filtered using a 0.2-µm filter. One hundred microlitres of supernatant was taken and vacuum-dried at 4 °C. The dried leftover was dissolved in 10 µL of freshly prepared methoxyamine hydrochloride solution (40 mg mL^−1^ in pyridine) and incubated at 30 °C for 90 min with shaking. To the above solution, 90 µL of *N*-methyl-*N*-(trimethylsilyl)trifluoroacetamide was added and incubated at 37 °C for 30 min. The samples were centrifuged at 14,000×*g* for 3 min, and the supernatant was taken for the GC/MS analysis. The samples were run on GC–MS/MS, and the data were analysed using MetaboAnalyst 4.0 (http://www.metaboanalyst.ca) [115].

### Statistical analysis

All the experiments were done in biological triplicates, and the mean of three values was used to calculate standard deviation (SD). The final data were represented as mean ± SD (denoting SD as the experimental error). Graphs were plotted using MS Excel software (Microsoft Corporation, USA).

## Data Availability

All data generated or analysed during this study have been provided in this manuscript.

## Abbreviations

N-: nitrogen deprivation
P-: phosphorous deprivation
S-: sulphur deprivation
TAGs: triacylglycerols
siROS: specific intracellular reactive oxygen species
PAR: photosynthetically active radiation
RPM: rotation per minute
TLC: thin-layer chromatography
FA: fatty acid
FAMEs: fatty acid methyl esters
SFAs: saturated fatty acid
PUFAs: polyunsaturated fatty acid
MUFAs: monounsaturated fatty acid
TCA: tricarboxylic acid
NAD(P)H: nicotinamide adenine dinucleotide phosphate
C4: Hatch–Slack pathway of CO_2_ fixation
ACCase: acetyl coenzyme A carboxylase
ATP: adenosine triphosphate
ADP: adenosine diphosphate
NOx: nitrogen oxides
